# The Weight of Comorbidities in the Specific Treatment of ATTR-Related Amyloid Cardiomyopathy

**DOI:** 10.1007/s11886-025-02308-6

**Published:** 2025-10-25

**Authors:** Anna Cantone, Marco Maria Dicorato, Aldostefano Porcari

**Affiliations:** 1 Cardiologic Centre, University of Ferrara, Cona, Italy; 2 Interdisciplinary Department of Medicine, University of Bari “Aldo Moro”, Polyclinic University Hospital, Bari, Italy; 3 Centre for Diagnosis and Treatment of Cardiomyopathies, Cardiovascular Department, Azienda Sanitaria Universitaria Giuliano-Isontina (ASUGI), University of Trieste, Trieste, 34149 Italy; 4 European Reference Network for Rare, Low Prevalence and Complex Diseases of the Heart (ERN GUARD-Heart), Trieste, Italy; 5 National Amyloidosis Centre, Division of Medicine, University College London, Royal Free Campus, Rowland Hill Street, London, NW3 2PF UK

**Keywords:** Transthyretin amyloid cardiomyopathy, Heart failure, Comorbidities, Disease-modifying treatments, Clinical management, Prognosis

## Abstract

**Purpose of Review:**

This review aims to provide an updated overview of the clinical management of heart failure and comorbidities in transthyretin amyloid cardiomyopathy (ATTR-CM). We sought to address key unanswered questions and current uncertainties regarding treatment response, prognosis, and optimization of care in this complex population.

**Recent Findings:**

Once considered rare, ATTR-CM is now increasingly recognized due to greater awareness and the possibility of non-invasive diagnosis. Patients are often identified at earlier stages, with lower mortality than historically observed. Disease-modifying therapies with proven efficacy in randomized trials are now available, yet many patients experience disease progression. In real-world practice, ATTR-CM patients are typically older and have multiple cardiac and extracardiac comorbidities, often representing exclusion criteria of clinical trials, which may influence treatment response and efficacy.

**Summary:**

Modern management of ATTR-CM should integrate heart failure treatment with tailored approaches to comorbidity care. Earlier diagnosis, real-world evidence, and strategies for patients outside trial populations will be essential to improve prognosis and guide future research.

## Introduction

Transthyretin amyloid cardiomyopathy (ATTR-CM) is a progressive, life-threatening cardiomyopathy caused by the accumulation of misfolded transthyretin protein in the form of amyloid fibrils within the myocardial extracellular space [1]. Disease occurs when amyloid deposits disrupt cardiac structure and function, leading to progressive heart failure (HF) symptoms, recurrent hospitalization and ultimately death. The sporadic, noninherited, wild type form (ATTRwt-CM) is a condition of older, predominantly male individuals, whereas the hereditary form (ATTRv-CM) can present earlier in life with a varying clinical phenotype, often comprising both restrictive cardiomyopathy and polyneuropathy [2].

Major advances in cardiac imaging and heightened disease awareness among clinicians have transformed the epidemiology of ATTR-CM, enabling diagnosis at earlier stages [3, 4]. At the same time, deeper understanding of disease pathophysiology has driven the development of specific disease‑modifying therapies, now available or in late‑stage clinical trials. These include three major classes: TTR stabilizers, TTR gene silencers (siRNA, ASO, and CRISPR-Cas9–based gene editing), and amyloid depleters (antibody-mediated clearance of fibrils) [5].

While these drugs have shown substantial benefits in clinical trial populations [6, 7], the clinical phenotype of real-world patients may be more challenging; patients often present with multiple comorbidities—cardiac and extracardiac—that can influence prognosis, treatment response and long-term net clinical benefit.

**Fig. 1:**
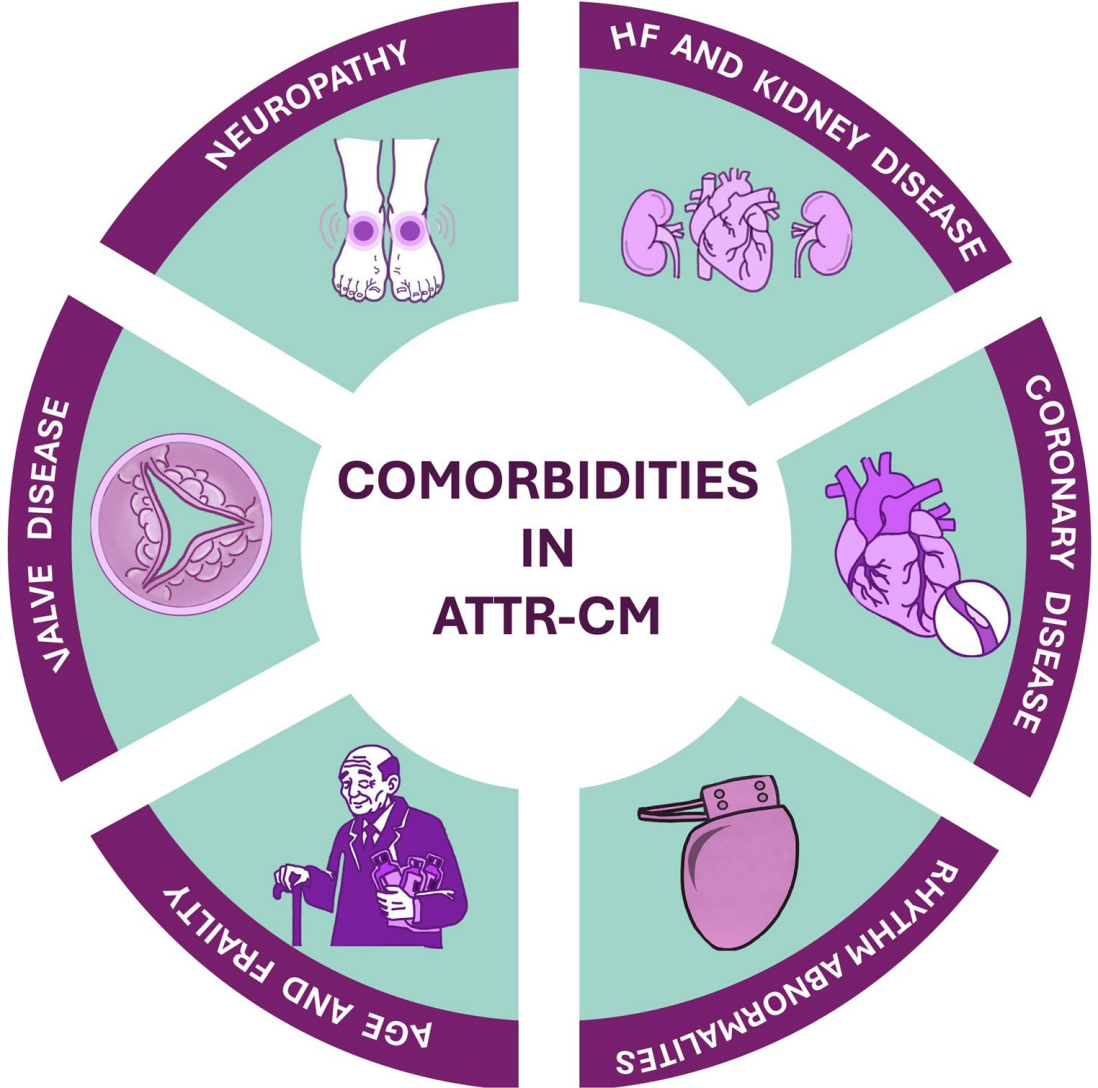
Cardiac and Extracardiac Comorbidities in Patients with ATTR-CM.**Key**: ATTR-CM, Transthyretin Amyloid Cardiomyopathy; HF, Heart Failure

This review will examine the weight of comorbidities in the specific treatment of ATTR-CM and will discuss how integrating comorbidity management into treatment strategies may improve patient outcomes and guide future therapeutic approaches **(**Fig. 1**)**.>

## Heart Failure and Kidney Disease

Historically, patients with ATTR-CM presented in advanced stages of infiltration, with a clinical picture dominated by right ventricular failure and peripheral congestion, manifesting as dyspnea, lower extremity oedema, jugular vein distention, hepatic congestion and ascites [1, 8]. Consequently, management has traditionally focused on supportive therapy, with loop diuretics representing the mainstay to achieve volume control [1, 8]. However, careful titration is essential as excessive diuresis can reduce preload, leading to organ hypoperfusion and worsening renal function [9].

Studies on conventional anti-neurohormonal therapies - such as beta-blockers (BBs), angiotensin-converting enzyme inhibitors (ACEi), angiotensin receptor blockers (ARBs), and mineralocorticoid receptor antagonists (MRAs) – have yield controversial results. Some suggested that low doses are well tolerated [10, 11], whereas others reported poor tolerance and even worse outcomes [12, 13].

Expert consensus recommendations generally advise avoiding (or caution use) ACEi/ARBs due to the risk of hypotension, discontinuing BBs in patients with low cardiac output, and provide conflicting recommendations for the use of MRAs [14]. Notably, most of these recommendations are based on historical cohorts with advanced disease and severe restrictive physiology. Contemporary patients are increasingly diagnosed in earlier disease stages, often before the onset of marked restriction. Emerging evidence indicates that neurohormonal activation in ATTR-CM may be similar to, or even greater than that seen in HF of different aetiologies [4, 15, 16], raising the possibility of benefit from neurohormonal modulation also in this patient population.

In real-world practice, BBs are often prescribed at low doses, mainly for concomitant atrial fibrillation (AF) and ischemic heart disease (IHD), and discontinued in about 20% of patients. ACEi/ARBs are generally prescribed for concomitant hypertension or proteinuric kidney disease, but are stopped in about one-third of cases. In contrast, MRAs are the most commonly tolerated drugs, with a discontinuation rate of only 8% [11, 17], likely reflecting their minimal impact on blood pressure and possible synergistic effect with loop diuretics.

Observational data suggest that low-dose BBs may reduce all-cause mortality in patients with a LVEF ≤ 40%, even after exclusion those with IHD. BBs have an established role in the treatment of HF with reduced ejection fraction in which have the ability to counteract the activation of adverse adrenergic and neurohormonal molecular pathways. However, BBs must be used cautiously, particularly in patients at high risk for bradyarrhythmias and conduction system disease [11, 17–19]. Treatment with MRAs has been associated with a lower risk of all-cause mortality across the full spectrum of LVEF, with the greatest benefit observed in patients with a LVEF >40%, suggesting they may be more beneficial in earlier stages of disease [9, 17].

Sodium-glucose cotransporter-2 inhibitors (SGLT2-i) have also shown encouraging data [20–22]. These drugs are generally well tolerated, with discontinuation rates around 10% or less, and have been associated to HF stabilization, lower diuretic need, and lower rates of cardiovascular mortality and HF hospitalization, regardless of concomitant disease-modifying treatment**(**Table 1**)**. Interestingly, SGLT2-i use was also associated with slower decline in renal function [20–22].

**Table 1:** Knowledge and uncertainties in the management of ATTR-CM

	Knowledge	Uncertainties
Heart Failure	- Loop diuretics for meticulous fluid control remain essential to improve HF symptoms. - Anti-neurohormonal agents are associated with lower mortality. - Consider low-dose β-blockers in LVEF ≤ 40%, and MRAs and SGLT2 inhibitors across the LVEF spectrum to improve outcomes.	- At what disease stage are HF medications most effective? - Do pre-symptomatic patients with established ATTR-CM benefit from these treatments? - Is there an impact on disease progression? - Will these agents remain effective in patients treated with disease-modifying or combination therapies?
Chest Pain and CAD	- CAD should be regularly assessed, and modifiable risk factors treated. - Treating significant stenosis of main epicardial coronaries improves prognosis but may not relieve symptoms due to microvascular dysfunction. - Low cardiac output syndrome is frequent after CABG.	- Should PCI be preferred over surgery? - How to manage antithrombotic therapy? - How to predict revascularization benefit on symptoms/prognosis, and address microvascular dysfunction?
Arrhythmias and Conduction Disease	**AF**- Rate control improves HF symptoms; low-dose β-blockers generally safe, digoxin should be avoided.- AV node ablation with biventricular pacing may be an option when other strategies fail.- Rhythm control: exclude cardiac thrombosis before cardioversion; AF recurrence is frequent; amiodarone preferred; catheter ablation possible but with limited/controversial evidence.	**AF** - Prognostic impact of rate vs. rhythm control? - Optimal drug selection? - When to prefer rate over rhythm control? - Does maintaining SR improve symptoms? - Are markers of atrial remodelling reliable to predict AF recurrence after cardioversion? - When to perform catheter ablation?
	**Ventricular arrhythmias and devices** - **ICD**: No proven survival benefit in ATTR-CM; secondary prevention can be considered in AL or hereditary ATTR-CM. Prophylactic ICD remains controversial, but may be used as bridge to transplant or in ischemic heart disease with standard indications.	**Ventricular arrhythmias and devices** -**ICD:** Which parameters should guide ICD implantation?- Can patients with biventricular dysfunction benefit from ICD?- How to predict SCD risk with shockable rhythm to guide prophylactic ICD?- Should ATTR and AL have different SCD prevention strategies?
	- **CRT**: LBBB is common at diagnosis; LVEF decline is a late finding. No defined CRT indications, but may benefit PM-dependent patients or those with RV pacing > 40%.	- **CRT**: Is CRT effective in ATTR-CM per HF guideline criteria?- Should CRT be offered based on HF symptoms and LBBB, regardless of LVEF?- Could para-Hisian pacing be preferable to RV pacing?
Thromboembolism & Anticoagulation	- **AF**: Anticoagulation recommended regardless of CHA₂DS₂-VASc, with careful bleeding risk assessment	- **AF**: Should LAA closure be considered in patients with prohibitive bleeding risk?
	- **SR**: Advanced atrial dysfunction promotes embolic events; CHA₂DS₂-VASc ≥ 3 associated with higher thromboembolic risk in small observational studies.	- **SR**: Should anticoagulation be used in SR with advanced atrial dysfunction?- Should cardiac thrombosis be excluded in all patients?- How to identify best candidates?
Valve Diseases – Aortic Stenosis	- Untreated severe AS has independent prognostic impact. - AVR (preferably TAVI) improves outcomes compared to medical management alone. - Higher risk of persistent AV block post-procedure; PM may be required. - Combining AVR with ATTR-specific therapy yields best prognosis, comparable to lone AS.	- Are standard AS severity criteria/calcium scores reliable in low-flow states? - How to monitor progression and predict TAVI feasibility? - How to predict AV block risk post-procedure? - Optimal timing for valve intervention in dual pathology, and its interaction with disease-modifying therapy initiation?
Valve Diseases – Mitral Regurgitation	- MR progression worsens prognosis and is an independent predictor of all-cause death. - Early experience with percutaneous repair shows procedural success.	- Are conventional parameters sufficient to assess MR in ATTR-CM restrictive physiology? - Long-term outcomes of percutaneous repair in ATTR-CM? - Ideal candidates for intervention to avoid futility?
Peripheral and Autonomic Neuropathy	- Rapidly progressive length-dependent axonal neuropathy is common in hereditary ATTRv; milder forms may occur in ATTRwt. - Autonomic dysfunction is frequent in ATTRv. - Neuropathy progression may be slowed by gene silencers; gabapentinoids relieve neuropathic pain.	- How to predict impact of therapies on neuropathy severity? - Do systemic therapies improve connective tissue manifestations (e.g., carpal tunnel, spinal stenosis)? - What is the optimal standardized toolset to diagnose, stage, and monitor autonomic dysfunction?
Polypharmacy and Frailty	- ATTR-CM drives frailty via multisystem involvement; frailty predicts worse prognosis. - Frailty assessment enhances risk stratification when combined with traditional markers (e.g., NAC stage, NT-proBNP). - Multidisciplinary care and comprehensive frailty evaluation recommended for all patients.	- What is the optimal frailty assessment tool for ATTR-CM? - Can disease-modifying therapy plus targeted interventions reverse or slow frailty progression? - How to integrate frailty into clinical pathways for high-cost/high-burden therapies?

Key: *AL* Light Chain Amyloidosis, *ACE-i/ARBs *angiotensin-converting enzyme inhibitors/ angiotensin receptor blockers, *AF* Atrial Fibrillation, *AS *Aortic Valve Stenosis, *ATTR-CM *Transthyretin Amyloid Cardiomyopathy, *ATTRv* variant Transthyretin Amyloidosis, *AV* Atrio-Ventricular, *AVR* Aortic Valve Replacement, *β-Bs* Beta Blockers, *CAD* Coronary Artery Disease, *CABG* Coronary Artery Bypass Graft, *CRT* Cardiac Resynchronization Therapy, *DCCV* Direct Current Cardioversion,* HF* Heart Failure, *ICD* Implantable Cardioverter Defibrillator, *LAA* Left Atrial Appendage, *LBBB* Left Bundle Branch Block, *LF-LG* Low-Flow Low-Gradient, *LVEF *Left Ventricular Ejection Fraction, *MRAs* Mineral Receptor Antagonists, *PCI* Percutaneous Coronary Intervention, *PM* Pacemaker, *RV* Right Ventricle, *SR* Sinus Rhythm, *TAVI* Transcatheter Aortic Valve Implantation

The kidney is a key player in ATTR-CM: it may be affected secondarily by HF (cardio-renal syndrome [23]) or directly by amyloid deposition [24]. Amyloid-related kidney damage usually manifests with proteinuria progressing to nephrotic syndrome and chronic kidney disease (CKD) [25]. In ATTR amyloidosis, renal involvement has been reported in 30% of patients with early-onset hereditary forms associated with the Val50Met (p.V50M) variant, sometimes preceding neurologic involvement [26]. In wild-type form and non-V50M ATTRv amyloidosis, overt proteinuria is uncommon, though recent studies suggest kidney involvement - often in the form of progressive CKD without significant urinary abnormalities – may be under-recognised [26, 27].

In contemporary series, worsening renal function occurs in 25–40% of patients with ATTR-CM [28, 29]. Lower estimated glomerular filtration rate (eGFR) at baseline [30, 31] or a >20% decline within 12 months has been associated with poorer survival [32]. Consistent with an amyloid-related pathophysiology, recent data suggest that treatment with stabilizers [33] and gene silencers [34] may help prevent or delay renal function deterioration, potentially broadening the therapeutic indications for these agents.

## Chest Pain and Coronary Artery Disease

Typical or atypical chest pain is a common symptom in ATTR-CM, being reported in 20% to 40% of patients as initial presentation, sometimes associated with a clinical picture resembling acute coronary syndromes [35, 36]. Chest pain has been associated to more advanced cardiac amyloid infiltration with elevated concentrations of cardiac biomarkers, and both diastolic and systolic dysfunction [36].

The risk of significant coronary artery disease (CAD) in patient with ATTR-CM is usually elevated, reflecting the older age of most patients and the frequent coexistence of cardiovascular risk factors, including hypertension, smoking, diabetes, CKD, and dyslipidaemia. Obstructive epicardial CAD has been reported in approximately 25% of patients with cardiac amyloidosis, with the highest frequency among those with ATTR-CM, hypertension and dyslipidaemia [36].

Notably, chest pain can occur even in the absence of obstructive epicardial CAD, due to microvascular dysfunction—a universal feature of cardiac amyloidosis. Amyloid deposition in the perivascular space, vascular wall and the myocardial extracellular space disrupts the capillary architecture and compress coronary microvasculature, leading to myocardial ischaemia and chest pain [37]. Histopathological studies report significant intramural coronary amyloid deposits in up to two-thirds of patients, mostly with immunoglobulin light chain (AL) amyloidosis, often accompanied by focal microscopic evidence of ischaemic injury [36, 38]. Recent cardiac magnetic resonance studies have shown that stress myocardial blood flow is markedly reduced in both ATTR- and AL amyloidosis compared to both obstructive and non-obstructive CAD controls [39].

The prognostic impact of CAD in ATTR-CM is debated: some studies report no significant influence on outcomes [35], while others find CAD associated with an increased risk of HF hospitalization [36]. In the acute setting, ATTR-CM patients presenting with ST-elevation myocardial infarction (STEMI) have higher in-hospital mortality rates and complications (including life-threatening arrhythmias, cardiogenic shock and acute kidney injury requiring dialysis) compared to those without STEMI [40]. Data on the survival benefit of revascularization strategies, including percutaneous coronary intervention (PCI) and coronary artery bypass grafting (CABG), in cardiac amyloidosis are inconclusive [40], and there is a lack of data in contemporary cohorts receiving disease-modifying treatments.

Management of chest pain in ATTR-CM remains challenging. Low-dose beta-blockers are most frequently used, while calcium-channel blockers are generally avoided due to poor tolerance and risk of bradyarrhythmias. ACEi and ARBs may be considered in hypertensive patients but are often poorly tolerated outside this setting. Anti-anginal drugs such as ranolazine, nitrates and ivabradine have an established role in relieving myocardial ischemia and may be used with caution, but data on their efficacy in patients with ATTR-CM is scarce [41]. Future research is needed to clarify the epidemiology of chest pain in ATTR-CM, the relative contribution of obstructive CAD versus microvascular amyloid infiltration, optimal medical strategies, and the potential benefits of coronary interventions in the era of disease-modifying therapies.

## Arrhythmias and Conduction System Disease

Beyond HF, the natural history of ATTR-CM is characterised by the development of arrhythmias and conduction system disease. AF is the most common arrhythmia, affecting up to 50–70% of patients with ATTRwt-CM and 20–30% of those with ATTRv-CM [42, 43]. In ATTRwt-CM, the annual incidence of new-onset AF is estimated at 10–15% [44, 45]. The prevalence of AF tends to increase with advancing disease stage, but its prognostic impact remains uncertain [45–47].

Management of AF in ATTR-CM is often challenging and requires decisions in three key areas: (a) rate vs. rhythm control strategy, (b) pharmacological vs. electrical therapy (direct current cardioversion [DCCV] or AF ablation), and, (c) anticoagulation and thromboembolic risk assessment.

Rate control is the most common initial approach. Low-dose BBs are typically used to achieve lenient control (< 110 bpm). Although digoxin has historically been discouraged, recent studies suggest it may be safe if drug levels and renal function are closely monitored [48]. Amiodarone, particularly in oral form, is generally well tolerated and effective for rate control.

At present, there is insufficient evidence to recommend rhythm control over rate control in ATTR-CM, given the variable clinical benefits and high recurrence rates. In clinical practice, rhythm control is mainly pursued in patients with significant symptom burden or intolerance to rate control drugs [42, 47, 49]. Atrial thrombi are relatively common in ATTR-CM, even anticoagulated patients [50, 51]. Therefore, a strategy including pre-cardioversion transesophageal echocardiography to rule out intracardiac thrombosis before proceeding with pharmacological or electrical cardioversion is advisable [50]. Amiodarone remains the antiarrhythmic agent of choice, although dofetilide and propafenone may be considered, with close QT and renal monitoring [52].

Non-pharmacological strategies include DCCV, AF ablation and AV node ablation. Success rates of DCCV in ATTR-CM are comparable to non-amyloid patients (approaching 90–95%) [47, 50], but require higher energy and more attempts, and carry a greater risk of complications - particularly ventricular arrhythmias and bradyarrhythmias requiring pacemaker (PM) implantation [50]. AF recurrence rates are high, ranging from 61% at 30 days to 48–80% at one year, depending on considered populations and median follow up time [47, 50, 53]. Data on efficacy of AF catheter ablation is currently limited, but the procedure appears safe in small series [54], with some studies suggesting a reduced rate of HF hospitalizations [47]. If some prognostic benefit exists in treating AF in ATTR-CM, achieving rhythm control in early disease course may yield better outcomes. Emerging data also suggest that tafamidis might reduce the occurrence of AF, although confirmation in larger studies is needed [55]. When both rate and rhythm control fail, AV node ablation followed by PM implantation could be an option to relief symptoms.

In the 2023 European Society of Cardiology on cardiomyopathies [56], anticoagulation is indicated for all ATTR-CM patients with AF, regardless of the CHA2DS2-VASc score, which underestimates the risk of systemic embolism in these patients [57]. Anticoagulation may also be considered in sinus rhythm in the presence of spontaneous echo contrast, transmitral A wave < 20 cm/s or reduced atrial strain [58, 59]. There are no robust data on the optimal anticoagulant type; direct oral anticoagulants (DOACs) are reasonable first-line agents, with warfarin reserved for mechanical valves, significant mitral stenosis, or recurrent thrombosis despite DOACs. In patients with contraindications to anticoagulation [60], left atrial appendage closure is an option, with periprocedural risks similar to non-amyloid patients [61], although evidence for long-term prevention of systemic embolism remains limited.

Amyloid deposition also affects the conduction system with development of AV delays, most commonly presenting with prolongation of the PQ interval (i.e., first degree AV block) and intraventricular delays with bundle branch blocks, leading to a significant proportion of patients requiring permanent PM implantation. In contemporary series, about 10% of patients with ATTR-CM already has a permanent PM at the time of diagnosis and 11% undergo PM implantation during follow-up [19, 62, 63]. A wide QRS interval (>120 ms) has been independently associated with the development of high-grade AV block [62, 63]. In a recent multicentre study investigating baseline ECG predictors of PPM in AL-CM and ATTR-CM, history of atrial fibrillation (any type), a PQ interval >200 ms and a QRS interval >120 ms were associated with permanent PM implantation [19]. The absence of all three risk factors identified patients without need for PPM in the following 6 months with an accuracy of 91.8% and a negative predictive value of 92% [19].

Biventricular cardiac resynchronization therapy (CRT) has shown promising results [64], although electrocardiographic criteria are often unmet. This is mainly because the development of systolic dysfunction is a late phenomenon in ATTR-CM and the majority of patients do not fulfil criteria for CRT. First experiences of left bundle branch area pacing have been published, demonstrating improvement in HF symptoms and good safety profile [65].

Non-sustained ventricular tachycardia (VT) can be found in 27% of patients during routine monitoring, but this percentage increases up to 74% in patients with implanted devices [66].

The clinical significance and prognostic implications of ventricular arrhythmias in ATTR-CM are yet to be determined, but recent studies suggest an association with increased mortality [42, 67, 68].

Therefore, a history of non-sustained VT has been proposed as useful finding when considering CA patients for ICD implantation [66]. Although secondary prevention is a strong indication for ICD implantation in patients with HF, the role of prophylactic ICD implantation in ATTR-CM remains controversial and it is not usually recommended [69]. Varr et al. [66] from the Stanford Amyloid Center proposed to consider ICD implantation for primary prevention in patients with NYHA < IV, life expectancy >1 year and history of exertional syncope or documentation of VT (either non-sustained or sustained) on ambulatory Holter monitoring. However, studies to date failed to demonstrate a net survival benefit following ICD implantation for primary or secondary prevention, reasonably due to (a) frequent arrhythmias not amenable to defibrillation (i.e. pulseless electrical activity) representing the predominant cause of arrhythmic death, (b) higher defibrillation thresholds of infiltrated hearts, (c) significant proportion of non-cardiac death, and (d) advanced ATTR-CM at diagnosis carrying ominous prognosis [49].

## Aortic Valve Stenosis and Mitral Valve Regurgitation

Valvular heart disease (VHD) is a common finding in ATTR-CM, as amyloid protein can accumulate in all valvular structures, with up to 44% of patients presenting with significant VHD [70]. This association is so strong that VHD is increasingly considered a red flag for diagnosing ATTR-CM, with aortic valve stenosis (AS) and atrioventricular valve thickening being key red flags for cardiac infiltration identifiable by echocardiography. These findings have shifted the perception of ATTR-CM from a cardiomyopathy to either a myocardial or a valvular disease model. Beyond its diagnostic implications, VHD is also associated with prognosis, as the additional haemodynamic load can worsen outcomes and influence therapeutic strategies [70, 71].

The link between AS and ATTR-CM is increasingly recognised. Studies indicate that approximately 11% of patients with severe AS have underlying ATTR-CM, rising to 18% in those over 80 years [71]. The diagnostic challenge of this dual pathology stems from the overlapping clinical and echocardiographic features, which often lead to underdiagnosis of ATTR-CM. Systematic screening is recommended for AS patients, and tools have been proposed to help identify individuals who warrant further investigation for amyloidosis [71]. In patients with dual pathology, the prognostic burden associated with the presence of severe AS is independent from that of ATTR-CM, with a reported two-fold increase in all-cause mortality. In a study of >800 untreated patients with ATTR-CM, severe AS was independently associated with all-cause mortality, also after adjustment for NYHA class and the NAC ATTR stage [72]. Although patients with dual pathology have a more decompensated clinical phenotype at presentation, recent evidence suggests that treating both conditions can significantly improve outcomes [71, 73]. Aortic valve replacement (AVR), preferably with a transcatheter approach (TAVI) in this population, has been shown to provide a survival benefit [71]. Consequently, the presence of ATTR-CM should not preclude per se suitability for TAVR in patients with severe AS. In addition, a large-scale international registry demonstrated that the combination of AVR and disease-modifying therapy results in the most favourable prognosis, with survival rates comparable to those of patients with lone AS undergoing AVR, highlighting the importance of diagnosing and treating the underlying myocardial disease in addition to the valvular lesion [73].

Mitral regurgitation (MR) is also a frequent finding in ATTR-CM, and systematic screening in patients undergoing transcatheter edge-to-edge mitral valve repair has revealed a dual pathology in almost 12% of cases [74].

The pathophysiology of MR in ATTR-CM is multifactorial, involving direct amyloid infiltration of the valve apparatus, characterised by leaflet thickening, altered annulus geometry, and chordae shortening, as well as cardiac remodelling driven by restrictive physiology [15, 75].

The development of new valve insufficiency or worsening in MR (and also TR) is an independent predictor of worse outcomes [76, 77]. Dynamic changes in the degree of valve regurgitation (even mild changes) cause a decrease in stroke volume [78] which, despite small in absolute terms, is hemodynamically significant for the amyloid infiltrated heart, resulting in a substantial reduction in forward flow and cardiac output (because of associated chronotropic incompetence).

Management typically involves optimising HF medical therapy, but in cases of primary MR, or severe secondary MR refractory to medical management, a multidisciplinary heart team approach is crucial to weigh the risks and benefits of intervention. While data on interventions are more limited than for AS, initial experiences with percutaneous mitral valve repair have shown safety and procedural success [74].

## Peripheral and Autonomic Neuropathy

Neurological manifestations are a key clinical feature of ATTR amyloidosis, with a presentation that varies significantly between genotypes [79, 80]. While prominent neuropathy is a central characteristic of certain hereditary (ATTRv) variants, a milder, more indolent form may also affect up to 30% of patients with wild-type ATTR, although its direct attribution to amyloidosis in these cases is often uncertain [81].

The sensorimotor polyneuropathy typical of ATTRv is a rapidly progressive, length-dependent, axonal condition, often starting with early small-fiber involvement. The clinical presentation typically includes paresthesia, numbness, pain, and weakness, particularly in the lower extremities, which can lead to gait instability, falls, and functional impairment [82].

Other common entrapment syndromes with neurological manifestations associated with amyloidosis, such as bilateral carpal tunnel syndrome and lumbar stenosis or lumbosacral radiculopathy, are distinct from polyneuropathy but can cause a similar pattern of numbness, pain, and weakness and may be confused with it [83].

Autonomic dysfunction (dysautonomia) is another, particularly severe, feature of ATTRv [84]. Its manifestations, including orthostatic hypotension, gastrointestinal distress, urinary retention, and erectile dysfunction, serve as crucial “red flags” for diagnosis [84].

Management requires a dual approach that targets both the underlying disease process and its symptoms. For polyneuropathy associated with ATTRv, disease progression can be slowed by disease-modifying treatments such as TTR gene silencers, while for symptomatic relief of neuropathic pain, treatment often involves agents like gabapentinoids [69].

Managing autonomic dysfunction is especially challenging in patients with coexisting cardiac amyloidosis, as drug therapies for orthostatic hypotension carry risks of worsening fluid retention or hypertension in patients with heart failure and restrictive physiology [69]. This complex interplay necessitates close collaboration between neurologists, cardiologists, and rehabilitation specialists to effectively balance symptom control with cardiovascular stability.

## Advanced Age, Polypharmacy, and Frailty

ATTR-CM is a disease typically diagnosed in older adults [1]. With an aging population and improved diagnostic techniques, patients often present with multiple age-related comorbidities, some of which can be exacerbated by the disease itself.

The multimorbidity, common in this population, frequently leads to polypharmacy, increasing the risk of drug-drug interactions, adverse reactions, and non-adherence. Furthermore, age-related declines in kidney and liver function can affect drug metabolism, and certain commonly prescribed medications, such as non-dihydropyridine calcium channel blockers and digoxin, require extreme caution in ATTR-CM due to their potential negative inotropic or toxic effects. This necessitates a careful, multidisciplinary approach to medication review to reduce non-essential treatment burden and avoid iatrogenic harm.

Frailty, defined as a state of decreased physiological reserve and increased vulnerability to stressors [85], is highly prevalent in this population, with a prevalence as high as 75% [86]. This multidimensional syndrome extends beyond physical weakness encompassing impaired mobility, malnutrition, and cognitive and mood disorders, many of which are directly worsened by the systemic manifestations of ATTR amyloidosis [87]. A recent analysis investigating the value of frailty in 880 patients with ATTR-CM, identified an association between Clinical Frailty Scale (CFS) and higher mortality risk across all age groups, genotypes, and disease stages [85].

The disease’s characteristic infiltration of nerves and musculoskeletal tissues contributes directly to the frailty phenotype through conditions like carpal tunnel syndrome, lumbar stenosis, and muscle weakness [1]. This creates a deleterious bidirectional relationship: ATTR-CM actively drives frailty, while the presence of frailty is independently associated with more severe cardiac involvement and a significantly worse prognosis.

Therefore, the convergence of advanced age, polypharmacy, and frailty demands an integrated care model centred around a Comprehensive Geriatric Assessment [86, 88]. This assessment is crucial not only to optimize management and address reversible components of frailty, but also to guide patient-centred decisions about advanced therapies, helping to mitigate both ageism and clinical futility in this uniquely complex population.

## Clinical Implications of Comorbidities for Treatment Response

The therapeutic landscape for ATTR amyloidosis, particularly for ATTR-CM, has undergone a paradigm shift. Until recently, the mainstay of management for patients with ATTR-CM was supportive therapy using loop diuretics to aid meticulous volume control. Advances in understanding the molecular basis of transthyretin amyloid fibril formation have enabled the development of disease-modifying therapies with proven efficacy in randomised clinical trials [89].

However, real-world data show that up to one-third of patients with early ATTR-CM experience disease progression despite receiving tafamidis [90]. This gap between trial efficacy and real-world clinical impact reflects the interplay of amyloid-related and non-amyloid mechanisms driving clinical deterioration and disease progression in contemporary patients **(**Fig. 1**)** [91]. As life expectancy increases, clinicians are facing challenging scenarios rarely encountered in the pre-tafamidis era **(**Table 1**)**. Many patients present now with comorbidities or disease stages that would have excluded them from participation in clinical trials, and these factors can substantially influence the expected net benefit of therapies **(**Table 2**)**:

**Table 2:** Selected inclusion and exclusion criteria used in randomised trials in ATTR-CM

Trials	ATTR-ACT	ATTRIBUTE-CM	APOLLO-B	HELIOS-B
Inclusion criteria	18–90 years	18–90 years	18–85 years	18–85 years
	History of HF (≥ 1 HF hospitalization or clinical HF)	History of HF (≥ 1 HF hospitalization or clinical HF)	History of HF (≥ 1 HF hospitalization or clinical HF)	History of HF (≥ 1 HF hospitalization or clinical HF)
	IVS thickness > 12 mm	IVS thickness ≥ 12 mm	IVS thickness > 12 mm	IVS thickness > 12 mm
	NT-proBNP ≥ 600 ng/L	NT-proBNP ≥ 300 ng/L	NT-proBNP > 300 (> 600 if AF) and < 8500 ng/L	NT-proBNP > 300 (> 600 if AF) and < 8500 ng/L
	NYHA I-III	NYHA I-III	NYHA I-III (low risk)	NYHA I-III (low risk)
		Stable doses of CV medical therapy	Clinically stable, no CV-related hospitalizations < 6 weeks of study start	Stable treatment for ≥ 2 weeks
	≥ 100 m on 6MWT	≥ 150 m on 6MWT in 2 tests	≥ 150 m on 6MWT	≥ 100 m on 6MWT
Exclusion criteria	NYHA IV	-	NYHA IV or NYHA III with NAC stage 3 (high risk)	NYHA IV or NYHA III with NAC stage 3 (high risk)
	-	NT-proBNP ≥ 8500 ng/L	NT-proBNP ≥ 8500 ng/L	NT-proBNP ≥ 8500 ng/L
	-	-	PND score ≥ 3	PND score ≥ 3
	Abnormal liver function*	Abnormal liver function*	Abnormal liver function*	
	Creatinine clearance ≤ 25 mL/min	eGFR < 15 mL/min/1.73 m^2^	eGFR < 30 mL/min/1.73 m^2^	eGFR < 30 mL/min/1.73 m^2^
	CCB or digitalis	Non-dihydropyridine CCB	Non-dihydropyridine CCB	Non-dihydropyridine CCB

Key: *6MWT* 6-minute walking test, *ACS* acute coronary syndrome, *AL* light-chain amyloidosis, *ATTR* transthyretin amyloidosis, *CCB* calcium channel blocker, *CV* cardiovascular, *eGFR* estimated glomerular filtration rate, *HF* heart failure, *HIV* human immunodeficiency virus, *IVS* interventricular septum, *mBMI* modified body mass index, *MI* myocardial infarction, *NT-proBNP* N-terminal pro-B-type natriuretic peptide, *NYHA* New York Heart Association, *TIA* transient ischemic attack, *TTR *transthyretin, *VAD *ventricular assist device

*Abnormal liver function defined as any of the following: 1) Aspartate transaminase or alanine transaminase levels ˃2.0

× the upper limit of normal (ULN); 2) Total bilirubin ˃2 × ULN; 3) International normalized ratio (INR) ˃1.5 (unless patient is on anticoagulant therapy, in which case excluded if INR ˃3.5)

- **Renal dysfunction** is a prime example: in ATTR-ACT and other trials, a minimum eGFR threshold (>25–30 ml/min) was required for enrolment, leaving uncertainty regarding safety, efficacy, and optimal dosing in patients with advanced CKD;
- **Neuropathy**: tafamidis was evaluated in patients with polyneuropathy disability (PND) stage I, while patisiran, inotersen, vutrisiran, and eplontersen included patients with PND stages I–II [6]. Patients with more advanced stages of neuropathy generally did not demonstrate meaningful clinical benefit, likely due to irreversible nerve damage, highlighting the importance of early diagnosis and timely treatment initiation;
- **Functional status**: in ATTR-ACT, the greatest benefit of tafamidis was observed in NYHA class I–II patients, with attenuated effect in NYHA class III patients. Subsequent trials such (ATTRibute-CM, APOLLO-B, HELIOS-B) excluded patients with severe functional impairment or advanced HF [6]. However, the paradoxical increase in cardiovascular hospitalization rates among NYHA class III patients in the treatment arm of ATTR-ACT may reflect a longer survival during a more severe phase of the disease [92]. Whether these patients benefit from disease-modifying treatment in the real-world, and whether the goal should be symptom relief or survival, remains uncertain;
- **Medical background therapy for HF**: use of treatments now recognised as potentially beneficial in ATTR-CM (BBs, MRAs, SGLT2 inhibitors) varied considerably across trials and between treatment and placebo arms, potentially modifying the observed treatment effect;
- **Frailty**: given the advanced age of most ATTR-CM patients, a structured, multiparametric frailty assessment—ideally involving geriatric expertise—should be incorporated into treatment planning to guide realistic goals of care and to prevent overtreatment or futile interventions in end-stage disease.

## Conclusion and Future Directions

Therapeutic innovation has transformed the management of ATTR-CM, yet important gaps in knowledge remain. Earlier diagnosis is paramount to maximising the benefits of treatment. Despite increased awareness and the availability of non-invasive diagnostic tools, delayed recognition is still common and wider adoption of systematic screening in high-risk groups could help close this gap. Another priority is generating robust real-world evidence for populations underrepresented or excluded from clinical trials. Registries and pragmatic clinical trials can provide invaluable insights into treatment safety, effectiveness, and optimal sequencing of therapies in these patients. Combination therapy also warrants investigation: given the distinct mechanisms of TTR stabilizers, silencers, and amyloid depleters, additive or synergistic effects are plausible. Defining the best timing and combinations will be critical, particularly for patients who progress despite treatment. Addressing the high cost of therapies and ensuring equitable access remain global challenges. Care should be delivered within a multidisciplinary framework, including cardiology, neurology, geriatrics, nephrology, haematology and rehabilitation medicine, to address the multisystem nature of the disease, optimise comorbidity management, and align interventions with patient goals.

The future of ATTR-CM management lies in earlier detection, particularly in pre-symptomatic stages, personalised treatment strategies directed towards amyloidosis and comorbidities, and health systems prepared to deliver promptly these advances to all who may benefit.

## Key References

- Ioannou A, Massa P, Patel RK, Razvi Y, Porcari A, Rauf MU, et al. Conventional heart failure therapy in cardiac ATTR amyloidosis. Eur Heart J 2023;44(31):2893–907.
  - This landmark study was the first to demonstrate that beta-blockers and MRAs are not only well tolerated in ATTR-CM, but can also significantly improve survival in patients with heart failure.
- Porcari A, Cappelli F, Nitsche C, Tomasoni D, Sinigiani G, Longhi S, et al. SGLT2 Inhibitor Therapy in Patients With Transthyretin Amyloid Cardiomyopathy. J Am Coll Cardiol. 2024;83(24):2411–22.
  - This was the first study to show that SGLT2 inhibitors are safe, well tolerated, and associated with improved clinical outcomes in patients with ATTR-CM and heart failure. Uniquely, 220 ATTR-CM patients receiving SGTL2 inhibitors were propensity-score matched to 220 untreated ATTR-CM using 16 key clinical variables, ensuring robust comparison.
- Nitsche C, Dobner S, Rosenblum HR, Patel KP, Longhi S, Yilmaz A, et al. Cardiac transthyretin amyloidosis treatment improves outcomes after aortic valve replacement for severe stenosis. Eur Heart J 2025; doi: 10.1093/eurheartj/ehaf362/8154293
  - This pivotal analysis demonstrated that in patients with dual pathology (ATTR-CM and severe aortic stenosis), sequential treatment with TAVI followed by tafamidis yields the most favourable prognosis, achieving survival rates comparable to those with lone aortic stenosis undergoing AVR.

## Data Availability

No datasets were generated or analysed during the current study.
